# The Small Chemical Compound Repsox Potentiates Oct4-Driven Astrocyte-to-Neural Stem Cell Reprogramming via Notch1/Hes1/Smurf2 Pathway

**DOI:** 10.1007/s10571-026-01731-9

**Published:** 2026-04-24

**Authors:** Xiaoyu Ma, Zijian Liu, Yuqing He, Dandan Zhang, Peng Deng, Lin Li, Xin Li, Junping Li, Quanrui Ma, Hao Yang

**Affiliations:** 1 Department of Anatomy, Histology and Embryology, School of Basic Medicine, Ningxia Medical University, Yinchuan, 750004 Ningxia Hui Autonomous Region China; 2 Department of Anatomy, Basic Medical School Academy, Shaanxi University of Chinese Medicine, Xianyang, 712046 Shaanxi Province China; 3 Translational Medicine Center, Hong Hui Hospital, Xi’an Jiao Tong University, Xi’an, 710054 Shaanxi Province China

**Keywords:** Repsox, Astrocytes, Cell reprogramming, Oct4, Neural stem cells, Cell therapy

## Abstract

Central nervous system (CNS) injury is a prevalent and devastating neurological disorder characterized by progressive and irreversible neuronal loss, leading to persistent neurological deficits. Cell-replacement therapy using pluripotent neural stem cells (NSCs) offers considerable promise for treating CNS injury treatment. Our recent studies have demonstrated that mature astrocytes can be directly reprogrammed to a pluripotent state through specific stimuli. However, acquiring sufficient quantities of functional induced NSCs (iNSCs) derived from astrocytes for clinical applications remains challenging due to the low efficiency and instability in previous methodologies. Consequently, it is of critical importance to improve the reprogramming efficiency of astrocytes into iNSCs. In this study, we showed that the reprogramming of astrocytes into iNSCs via a single transcription factor Oct4 is significantly enhanced by continuous treatment with Repsox, a small molecule inhibitor of transforming growth factor-β (TGF-β) signaling. This enhancement was substantiated by increased efficiency in both reprogramming and conversion processes towards genuine NSCs, as demonstrated by the acquisition of distinctive hallmark NSC properties, including distinct morphological features, self-renewal capacity, expression of NSC-specific markers, and multipotency. Furthermore, the resulting iNSCs successfully differentiated into astrocytes, neurons, and oligodendrocytes. Notably, compared to iNSCs generated by Oct4 alone, Oct4/Repsox-induced NSCs exhibited a greater propensity to give rise to more neurons with neuronal functional properties, and relative fewer glial cells. Mechanistically, the activation of Notch1/Hes1/Smurf2 signaling cascades was involved in this enhanced intricate cell reprogramming events. The efficient reprogramming of astrocyte into iNSCs will provide a promising autologous cell-based therapeutic strategy for patients with CNS injury.

**Supplementary Information:**

The online version contains supplementary material available at 10.1007/s10571-026-01731-9.

## Introduction

The inevitable neuronal loss caused by the central nervous system (CNS) injury is particularly devastating, frequently resulting in severe and permanent neurological deficits in patients (Liau et al. 2020; Lv et al. 2021; Puls et al. 2020). Until now, no effectively curative treatment is available for SCI, and both its incidence and prevalence rates of SCI increasingly rise annually (Poulen et al. 2021; Leister et al. 2022). Nonetheless, NSCs have emerged as highly promising candidates for cell-replacement therapies to mitigate irreversible neuronal loss and ultimately restore neurological functions. Despite recent advances in promoting neural regeneration through the transplantation of various types of stem cells, there were still several formidable challenges, such as very limited cell sources, ethical concerns, immune rejection, and tumorigenicity, all of which significantly hinder the clinical application of stem cell transplantation in neural repair (Yamanaka et al. 2020; Xu et al. 2021). Encouragingly, emerging evidence suggests that somatic reprogramming technologies might address these hurdles by generating specific, therapeutically relevant cell types (Rosa et al. 2022; Wang et al. 2021; Gao et al. 2021; Bektik et al. 2021; Liu et al. 2021).

Glial cells play critical roles in neurophysiological processes, including neural activity performance and maintenance of homeostasis (Masuda et al. 2020; Kim et al. 2020; Sancho et al. 2021; Duan 2020; Araki et al. 2021). Among glial subtypes, astrocytes are the most abundant in the central nervous system (CNS) and are essential for its development and normal function (Hasel et al. 2021; Miyazaki et al. 2020; Linnerbauer et al. 2020; Fan et al. 2021). Recent studies have shown that astrocytes can revert to a primitive, undifferentiated state with characteristics resembling immature glial-like cells or neural precursor cells. This phenotypic transition can be triggered by diverse stimuli, including low temperature exposure, mechanical injury, ischemia, or direct reprogramming into neuron lineages (Chang et al. 2007; Shin et al. 2013; Lang et al. 2004). Notably, astrocytes demonstrate remarkable plasticity and can be reprogrammed into pluripotent stem cells, neural precursor cells, lineage-committed cell types, and specific neuronal subtypes via ectopic expression of defined factors or other interventions (Wei et al. 2021; Peng et al. 2022; Huang et al. 2020; Sharif et al. 2021; Steindler et al. 2003; Wang et al. 2015; Niu et al. 2015; Gao et al. 2017; Guo et al. 2014; Yang et al. 2016; Do et al. 2021). These induced cells can readily differentiate into diverse cellular phenotypes via various methodological approaches, and the resulting reprogrammed populations maintain stable biological characteristics and functions (Yang et al. 2016). Notably, a growing body of studies highlight astrocytes as one of the most amenable somatic cell types for reprogramming into closely related neural lineages. Importantly, they can be directly converted into induced neural stem cells (iNSCs) without the need for intermediate lineage stages or invasive grafts in clinical applications (Wang et al. 2015; Guo et al. 2014). Therefore, astrocytes have widely been regarded as highly promising endogenous candidates for developing cell-based therapies for CNS injury. Although astrocytes possess considerable potential, enhancing the efficiency of astrocyte reprogramming and minimizing incomplete reprogramming are crucial for the successful generation of astrocyte-derived NSCs and their effective translation into clinical application.

**Fig. 1:**
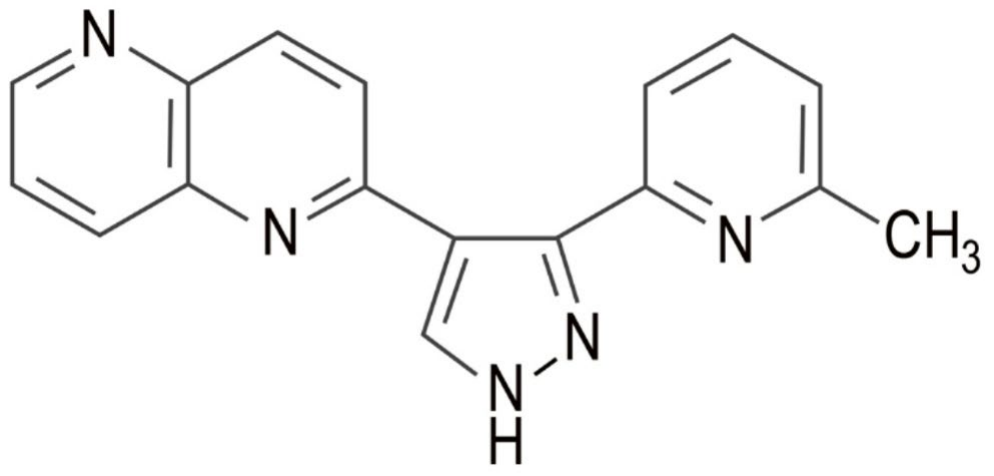
Schematic diagram of chemical structure of Repsox (2-(3-(6-methylpyridin-2-yl)-1 H-pyrazol-4-yl)-1,5-naphthyridine)

Despite significant progress in astrocyte-to-NSC reprogramming through ectopic expression of the transcription factors (TFs), current TF-based methods still rely predominantly on retroviral transduction of NSC-associated TFs, either individually or in combination. However, these approaches frequently fail to overcome several critical issues, including extended processing durations, tumorigenic risk, as well as inconsistent reprogramming efficiency and stability. To overcome these challenges, it is essential to develop optimal induction strategies that minimize the number of required TFs while incorporating supportive small molecules to enhance both efficacy and safety. Repsox (E-61645), a small molecule inhibitor of TGF-β receptor 1 (TGFβ1) kinase. The TGF-β superfamily regulates a wide range of cellular biological processes and plays a critical role in modulating cellular responses such as differentiation, proliferation, growth, adhesion, migration, survival, apoptosis, and developmental fate determination (Do et al. 2021; Ichida et al. 2009; Gellibert et al. 2004; Woltjenet al. 2009; Utikalet al. 2009). TGF-β signaling acts through both canonical Smad-dependent and non-canonical Smad-independent pathways (Gao et al. 2019; Derynck et al. 2003; Zhang et al. 2017). Notably, Repsox has been shown to functionally replace transgenic Sox2 in reprogramming systems, fulfilling its biological role in cellular physiological processes even in the absence of VPA and cMyc (Ichida et al. 2009; Maherali et al. 2009; Zhang et al. 2020). As illustrated in Fig. 1, the bioactive component of Repsox, 2-(3-(6-methylpyridin-2-yl)-1 H-pyrazol-4-yl)-1,5-naphthyridine, acts as a crucial upstream modulator of TGF-β signaling in multiple pathophysiological contexts, including cellular reprogramming (Guo et al. 2021a, b, c; Fu et al. 2015), osteosarcoma suppression, inhibition of osteoclastogenesis, bone resorption, and regulation of leukemic stem/progenitor cell development (He et al. 2021; Mei et al. 2018; Jajosky et al. 2014; Ide et al. 2017). Our prior in vitro study has shown that the TF Oct4, combined with sonic hedgehog (Shh), significantly enhances both the efficiency of reprogramming and the functional maturation of astrocyte-derived iNSCs (Yang et al. 2019). Mechanistically, this process appears to rely substantially on Sox2 activation and its downstream pathways, which play pivotal in orchestrating the complex astrocyte-to-NSC reprogramming. Based on these findings, we hypothesize that Repsox may function as a potent facilitator of astrocyte-to-NSC conversion, potentially improving not only the efficiency but also fidelity of this cellular conversion.

In this study, we developed in vitro astrocyte cultures to investigate Oct4-mediated reprogramming in the presence of Repsox, a small-molecule substitute for the key transcription factor Sox2. We found that ectopic expression of Oct4 alone was sufficient to reprogram astrocytes into iNSCs. Notably, the addition of Repsox significantly enhanced this TF-driven reprogramming efficiency. Of significance, the resulting iNSCs more closely resembled authentic NSCs in terms of both cellular morphology, gene expression profiles, and their capacity to differentiate into all major neural cell lineages in vitro and in vivo. Furthermore, neurons derived from these iNSCs exhibited characteristic neuronal properties, including synapse formation and electrophysiological activity. Mechanistically, Repsox administration significantly enhanced the efficiency of Oct4-mediated astrocyte reprogramming, primarily through activation of the Notch1/Hes1/Smurfs signaling pathways. Collectively, these findings propose a novel and efficient strategy for generating NSCs from autologous astrocytes, highlighting their potential for cell-based replacement therapy in CNS repair.

## Materials and Methods

### Main Reagents

Dulbecco’s modified Eagle’s medium (DMEM)/F12, N2 supplement, fetal bovine serum (FBS) and trypsin were purchased from Gibco (Carlsbad, CA, USA); basic fibroblast growth factor (bFGF), epidermal growth factor (EGF), poly-L-lysine (PLL), bovine serum albumin (BSA), DAPT and USF2 were purchased from Sigma-Aldrich (Merck KGaA Darmstadt, Germany); Repsox was purchased from MedChem Express (Shanghai, China); Lentivirus carrying the Oct4 overexpression gene (HBLV-r-Pou5f1-3xflag-ZsGreen-PURO) were purchased from Hanbio Biotechnology (Shanghai, China); mouse anti-GFAP antibody (Cat# ab4648, RRID: AB_449329), rabbit anti-Olig2 antibody (Cat# ab254043, RRID: AB_2927454), rabbit anti-Nestin antibody (Cat# ab221660, RRID: AB_2909415), mouse anti-MAP2 antibody (Cat#ab5392, RRID: AB_2138153), and rabbit anti-S100-β antibody (Cat# ab52642, RRID: AB_882426), and rabbit anti-CD133 (Cat#ab19898, RRID: AB_470302) were purchased from Abcam (Cambridge, MA, USA); mouse anti-Tuj1 antibody (Cat#70668, RRID: AB_2799825), rabbit anti-PSD-95 antibody (Cat#3409, RRID: AB_1264242), rabbit anti-SYN antibody (Cat#5297, RRID: AB_2616578), rabbit anti-Smurf2 antibody (Cat#12024, RRID: AB_2797800), rabbit anti-Hes1 antibody (Cat#11988, RRID: AB_2728766), and rabbit anti-Notch1 antibody (Cat#4380, RRID: AB_10691684) were purchased from Cell signaling Technology (Danvers, MA, USA); mouse anti-β-actin (Cat# HA-R1207-1-200, RRID: AB_3076050) was purchased from Liankebio (Hangzhou, Zhejiang, China); HRP-conjugated goat anti-rabbit (Cat#AS00039, RRID: AB_10746228), HRP-conjugated goat anti-mouse (Cat#GB23301, RRID: AB_2904020) were purchased from ZSGB-BIO (Beijing, China); Alexa Fluor 594 donkey anti-mouse lgG (H + L) (Cat#R37115, RRID: AB_2556543), Alexa Fluor 488 donkey anti-rabbit lgG (H + L) (Cat#35553, RRID: AB_1965947), and TRIZOL reagent were purchased from Life Technologies (Eugene, OR, USA); Cell counting kit-8 (CCK-8) was purchased from Boster Biological Tech ( Hubei, China); Revert Aid First strand cDNA synthesis kit and Power SYBR Green PCR Master Mix were purchased from Thermo Fisher Scientific, Inc (Waltham, MA, USA); All PCR primers were synthesized by Sangon Biotech (Beijing, China).

### Animals and Ethical Permission

All the experiments were conducted in full compliance with the National institutes of Health guidelines. The experimental protocol was approved by the Institutional Animal Care and Use Committee of Xi’an Jiaotong University (Approval Project Title: The roles of prolonged release of Shh signaling in Oct-4-induced de-differentiation of astrocytes to neural stem cells”, Approval No: 202003058, Approval date: Dec 04, 2020). All rats were housed in groups of six per cage in a controlled environment under a 12-h light/dark cycle and free access to food and water.

### Randomization and Blinding

To minimize selection bias and ensure baseline comparability across experimental groups, computer-generated random numbers were used for group allocation. To maintain objective, each animal was assigned a unique identifier that corresponded to the randomization sequence by a researcher not involved in any aspect of the experimental procedures, Additionally, a double-blind design was implemented: both the experimenter and data analysts remained unaware of group assignments until the completion of the final analysis, ensuring unbiased data collection and strengthening result credibility.

### Isolation and Culture of Cortical Astrocytes

Primary astrocyte cultures were prepared from the cerebral cortices of 3-day-old neonatal Sprague-Dawley rat pups as previously described (Kogel et al. 2021; Shi et al. 2013). Briefly, postnatal day 3 (P3) rat pups were anesthetized with 5% isoflurane in a sealed induction chamber for approximately 2 min. Upon confirmation of deep anesthesia (assessed by the absence of pedal reflex), rapid decapitation was performed using sterile surgical scissors. The rat heads were sterilized by immersion in iodine solution for 1 min, followed by a rinse with 75% ethanol. The brains were promptly extracted, and bilateral cortices were isolated under sterile conditions. Cortical tissues were enzymatically dissociated into single-cell suspension, and plated onto PLL-coated flasks at a density of 2 × 10^6^/ml. Cells were maintained in DMEM/F12 medium supplemented with 10% fetal bovine serum (FBS) and incubated at 37 °C in a humidified atmosphere of 5% CO2/95% air incubator. The culture medium was refreshed once every 3 days. After 7 days in culture, astrocytes were purified utilizing differential cell adhesiveness (Yang et al. 2019). The identity and purity of the isolated astrocytes were subsequently verified by immunolabeling for the markers GFAP, S100β, and Nestin.

### Production of Lentiviral Particles Expressing Oct4

The rat *Oct4* cDNA sequence was subcloned from the HBLV-r-Pou5f1-3xflag-ZsGreen-PURO plasmid. To generate Oct4-expressing lentiviral particles, the cDNA fragment was amplified by high-fidelity PCR and subsequently cloned into a PGMLV-based lentiviral vector, following the protocol described by Yang et al. (Yang et al. 2019; Lin et al. 2021). The constructed PGMLV-Oct4 plasmids was verified by sequencing prior to lentiviral packaging. For virus production, the plasmid was transfected into 293T cells (The cell line was originally established from human embryonic kidney cells through transfection with the SV40 T antigen gene and was obtained from the Institute of Cell Biology, Chinese Academy of Sciences. Its genetic identity was confirmed by short tandem repeat (STR) profiling performed by the same institute, which verified that the cell line matched the expected genetic profile. Cells were cultured in Dulbecco’s Modified Eagle Medium (DMEM) supplemented with 10% FBS and were passaged using 0.25% trypsin-EDTA upon reaching 80–90% confluence. Following detachment, cells were reseeded at a split ratio of 1:4 to 1:8, as recommended by the manufacturer) using Lipofectamine 3000. Viral supernatant was collected 36–48 h post-transfection, filtered through a 0.45-µm syringe filter, and concentrated by ultracentrifugation to obtain high-titer viral stocks. Finally, the concentrated virus was aliquoted (50 µL/tube) and stored at − 80 °C until further use.

### Conversion of Astrocytes into Induced Neural Stem Cells (iNSCs)

For in vitro reprogramming, mature passage 1 (P1) astrocytes were seeded at a density of 1 × 10^6^ cells/ml in PLL-coated 6-well plates. Upon reaching approximately 85% confluency, they were transduced with Oct4 lentivirus produced as previously described (Yang et al. 2019). After 24 h of transduction, the viral supernatant was refreshed with DMEM/F12 medium supplemented with 1% G5. Two days later, the medium was switched to NSC medium, consisting of DMEM/F12 medium, 2% N2, 20 ng/ml bFGF, and 20 ng/ml EGF. On day 4 post-infection, Repsox was introduced at a final concentration of 25 µM and maintained continuously for 7, 10, and 14 days. Subsequently, the resulting floating neurospheres were collected, dissociated enzymatically with Accutase, and resuspended in NSC medium for subculture at 1 × 10^6^ cells/ml in 60 mm low-adhesion dishes. Neurosphere identity was further confirmed by immunostaining for Nestin. To explore molecular mechanisms underlying the reprogramming, two inhibitors DAPT (a Notch1 inhibitor, 2 µM) and USF2 (a Smurf2 inhibitor, 1 µg/mL), were introduced into the reprogramming system in various combinations and maintained for 2–3 weeks before subsequent experiments.

### Enhanced Generation of iNSCs by the Small-Molecule Compound Repsox

To assess neurospheres formation from reprogrammed astrocytes under two experimental conditions, 1** × **10^5^ cells were transfected with Oct4 lentivirus and treated with or without Repsox for different durations. Following transduction, the cells were maintained in NSC medium in low-attachment 6-well plates for two weeks. Neurospheres were quantified by averaging counts from at least two of three independent replicate wells per sample. Briefly, all floating neurospheres were collected by centrifugation, replated onto PLL-coated glass coverslips, and allowed to settle for 30 min. Neurospheres within 15 randomly selected fields of view (each 0.45 mm²) were then counted. Only neurospheres with diameters between 50 and 100 μm or ≥ 100 μm were included in the count analysis. To evaluate the self-renewal capacity, these neurospheres were further subcultured for five passages following the aforementioned procedure. All counts were performed by an investigator blinded to experimental conditions to minimize bias. Cell quantification and subsequent analysis were conducted as previously described (Ichida et al. 2009; Favaro et al. 2009).

### qRT-PCR Analysis

Total RNA was extracted from iNSCs under various treatments using TRIZOL reagent. Subsequently, 2 µg of purified total RNA was reverse-transcribed into cDNA using a Revert Aid First Strand cDNA Synthesis kit, according to the manufacturer’s instructions. Quantitative real-time PCR (qRT-PCR) was carried out using Power SYBR Green PCR Master Mix to measure mRNA expression levels of the following target genes: GFAP, S100β, Nestin, CD133, Pax6, Sox2, Nanog, PCNA, and CyclinD1. GAPDH was used as the endogenous control for normalization. The primer sequences for the gene analysis are listed in Table 1. qRT-PCR was conducted in accordance with the manufacturer’s recommended instructions. Data were processed with CFX Manager™ 3.1 software (Bio-Rad, USA). Relative mRNA levels were calculated with the standard ΔΔCt method. All data are presented as Mean ± SD. Statistical analysis (*p* < 0.05) was performed using unpaired Student’s t-test for comparisons between two groups, and one-way ANOVA followed by Tukey’s post-hoc test for multiple comparisons. A p-value of less than 0.05 was considered statistically significant. All experiments included at least three independent biological replicates for each sample to ensure reliability and reproducibility of the results.

### Neuronal and Glial Differentiation of iNSCs

To evaluate the differentiation potential of astrocyte-derived NSCs into neuronal and glial lineages, passage-2 (P2) neurospheres were plated on PLL-coated coverslips in a 24-well plate at a density of 30 neurospheres per coverslip. To induce differentiation, iNSCs were maintained in a DMEM/F12-based medium supplemented with 2% fetal bovine serum (FBS) and 2% B27 for 9 days, with half of the differentiation medium replaced every 3 days. Subsequently, immunofluorescence staining and western blot analysis were conducted to assess iNSC differentiation.

To assess neuronal differentiation potential of astrocyte-derived NSCs in vivo, twenty adult SD rats weighing approximately 250 g were used as recipients and randomly assigned to four groups as follows: Con group (transplantation of astrocyte); Repsox group (Repsox-treated astrocyte transplantation); Repsox+Oct4 group (transplantation of NSCs derived from Repsox/Oct4-treated astrocyte), and Oct4 group (transplantation of NSCs derived from Oct4-treated astrocytes). Cell suspension preparation and cell transplantation procedure was conducted according to our previously described (He et al. 2023; Guo et al. 2021a, b, c). Briefly, after anesthesia and the establishment of animal models without spinal cord damage, cells were pre-labelled with hoechest33342. A total of 1 µl mixed cell suspension at density of 1 × 10^5^ /µl) was slowly injected into spinal cord using a microsyringe. During the first three postoperative days, assisted micturition was performed twice daily, and penicillin was administered intraperitoneally to each rat to prevent urinary tract infection.

### Immunofluorescence Staining

Primary astrocytes, iNSCs, and other treated cells were fixed with 4% paraformaldehyde for 15 min at room temperature. After three washes with PBS, cells were blocked with 1% bovine serum albumin (BSA) for 1 h at room temperature. Subsequently, the samples were incubated overnight at 4 °C with the following primary antibodies: mouse anti-GFAP (1:200), rabbit anti-Olig2 (1:200), rabbit anti-Nestin (1:200), rabbit anti-S100β (1:200), rabbit anti-GFAP (1:200), and mouse anti-Tuj-1 (1:200). Following removal of the primary antibodies and three thorough washes with PBS, the cells were incubated with appropriate fluorescein-labeled secondary antibodies (Alexa Fluor 594 donkey anti-mouse lgG (H + L), 1:500; Alexa Fluor 488 donkey anti-rabbit lgG (H + L) 1:400) for 1 h in the dark at room temperature. Nuclei were then counterstained with DAPI for 10 min. For immunofluorescence of the spinal cord sections, section preparation and staining procedures followed previously described methods (He et al. 2023). Finally, the coverslips were mounted onto glass slides, and positive cells was visualized and quantified using a fluorescence microscope (Zessis LSM880 Laser Scanning microscopes) equipped with a 10× or 20× objective lens. The following settings were applied: a white light laser was used for excitation at 488 nm and 594 nm. Emission light was collected using hybrid detectors (HyD) with gaAsP detectors within ranges of 500–550 nm and 560–620 nm, respectively.

### Western Blot

Protein from each group were extracted by lysing cells in ice-cold buffer supplemented with protease inhibitors for 30 min. The protein homogenates were subsequently centrifuged at 12,000 rpm for 10 min at 4 ˚C. The resulting supernatants were collected and subjected to western blot analysis following previously described protocols (Yang et al. 2019; Ding et al. 2016). The following primary antibodies were used: GFAP (1:1000), Olig2 (1:500), Tuj-1 (1:1000), PSD-95(1:800), SYN(1:1000), Notch1(1:1000), Hes1(1:800), Smurf2 (1:1000), Nestin (1:1000),, and CD133 (1:800), with β-actin (1:1000) serving as the loading control. After three thorough washes with PBS, the membranes were incubated with secondary antibodies (goat anti-rabbit, 1:5000; goat anti-mouse, 1:5000). the immunoblots were visualized using enhanced chemiluminescence and imaged with a gel system camera. The relative protein expression levels were normalized by calculating the ratio of the target protein (GFAP, Olig2, Tuj-1, PSD-95, SYN, Notch1, Hes1 Smurf2, Nestin, and CD133) to β-actin.

### Electrophysiology

After three weeks of induction period, the astrocyte-derived NSCs were differentiated and transferred to artificial cerebrospinal fluid (ACSF) continuously oxygenated with 95% O₂/5% CO₂ for electrophysiological properties. Whole-cell patch-clamp recordings were performed at 20–22 °C under an inverted microscope (Zeiss, Jena, Germany), as described previously (Yang et al. 2019).

### Statistical Analysis

In all analyses, data are presented as mean ± SD from at least three independent experiments. Statistical analysis were preformed using Statistics Data Editor 17.0 (SPSS, Inc., Chicago, IL, USA). Differences among multiple groups were assessed by One-way ANOVA, while pairwise comparisons between two groups were conducted using an independent-Sample t-test. A value of *p < 0.05 was defined as statistically significant.

## Results

### Characterization and Identification of Primary Astrocytes

**Fig. 2:**
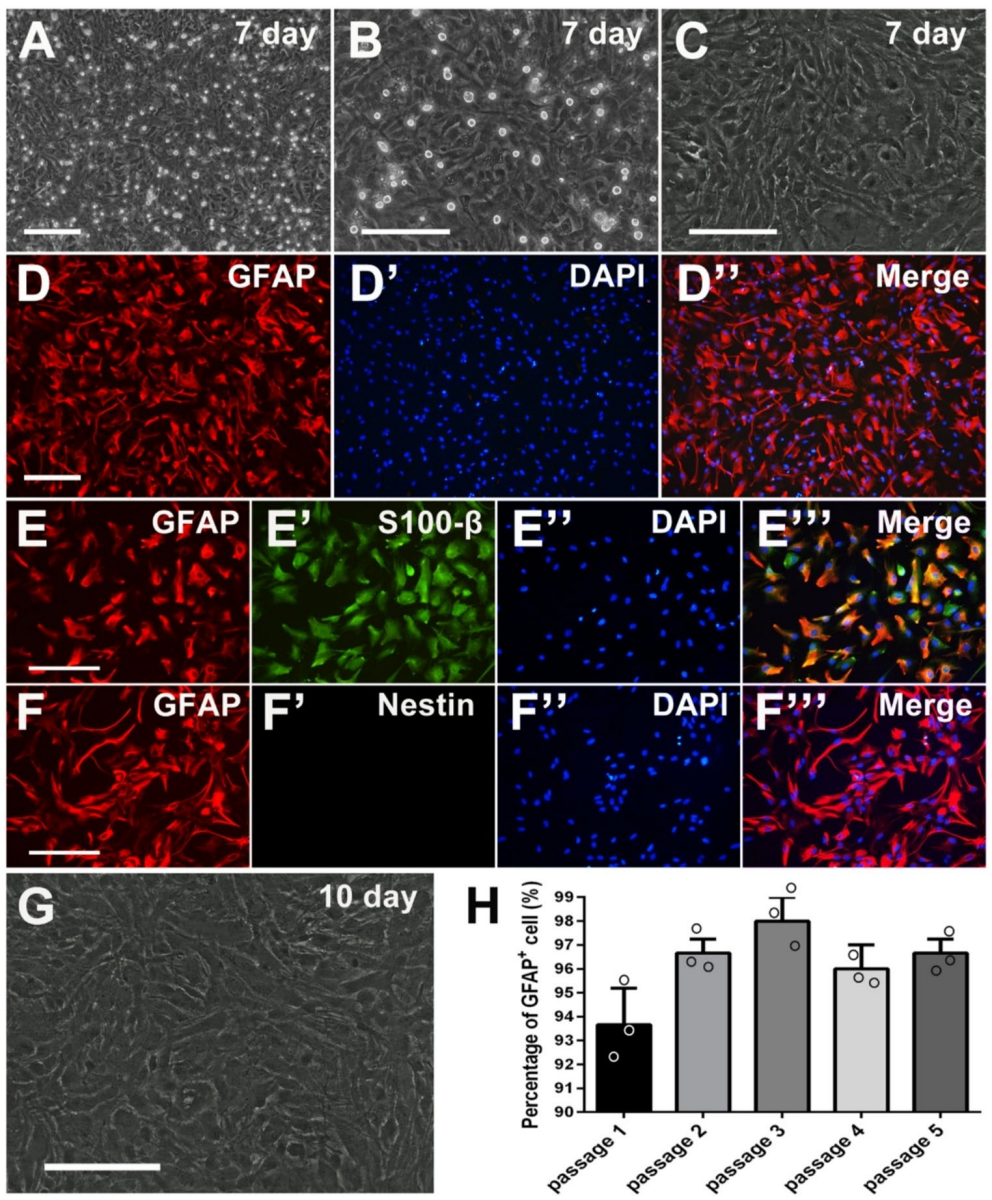
Morphological and biochemical characteristics of primary astrocytes. (**A**,** B**) phase-contrast micrographs of primary astrocytes on day 7 at 10 × and 20 × magnification, respectively. (**C**,** G**) Phase-contrast images of purified astrocytes on day 7 and 10 under. (**D**─**D’’**) Representative immunofluorescence photomicrographs showing GFAP expression. (**E**─**E’’’**) Double –immunostaining for GFAP (red) and S100-β (green) in purified astrocytes. (**F**─**F’’’**) Immunostaining for GFAP (red) and Nestin (green) in the cells. (**D’’**,** E’’**, and** F’’**) Corresponding images with DAPI nuclear counterstaining. (H) Percentages of GFAP-immunoreactive cells across subcultures from passage 1 to passage 5. All data are presented mean ± SD and are representative of three independent experiments with similar results. Scale bars: 100 μm

To investigate whether Repsox enhances Oct4-mediated astrocyte-to-iNSC reprogramming, primary astrocytes were first isolated from rat cortices, cultured, characterized, and identified. As shown in Fig. 2A-B, phase-contrast microscope at day 7 revealed that the cells predominantly displayed the typical flat, polygonal morphology of astrocytes and formed a confluent monolayer. Within this monolayer, a subset of cells exhibited small somas and short processes. After purification, cultures adopted homogeneous astrocytic morphology and reached > 95% cell confluence (Fig. 2C, G). Immunophenotypic validation was conducted using the astrocytic markers GFAP and S100β, as well as the neural precursor marker Nestin. The purified cell population showed strong and ubiquitous positive for both GFAP and S100β (Fig. 2D’’, 2E’’ and 2E”’), indicating their terminally differentiated astrocytic identity. Of crucial importance, these GFAP-positive cells entirely devoid of Nestin immunoreactivity (Fig. 2F”’), thereby effectively ruling out contamination by neural precursor cells. Quantitative analysis further supported these observations, demonstrating that the cultures maintained high purity (more than 90% GFAP-positive astrocytes) over at least five successive passages (P1: M = 93.85, SD = 1.55; P2: M = 96.71, SD = 0.53; P3: M = 97.91, SD = 0.81; P4: M = 96.1, SD = 0.63; P5: M = 96.75, SD = 0.45) (Fig. 2H).

### Repsox Enhances Oct4-Driven Reprogramming of Astrocytes into NSCs

**Fig. 3:**
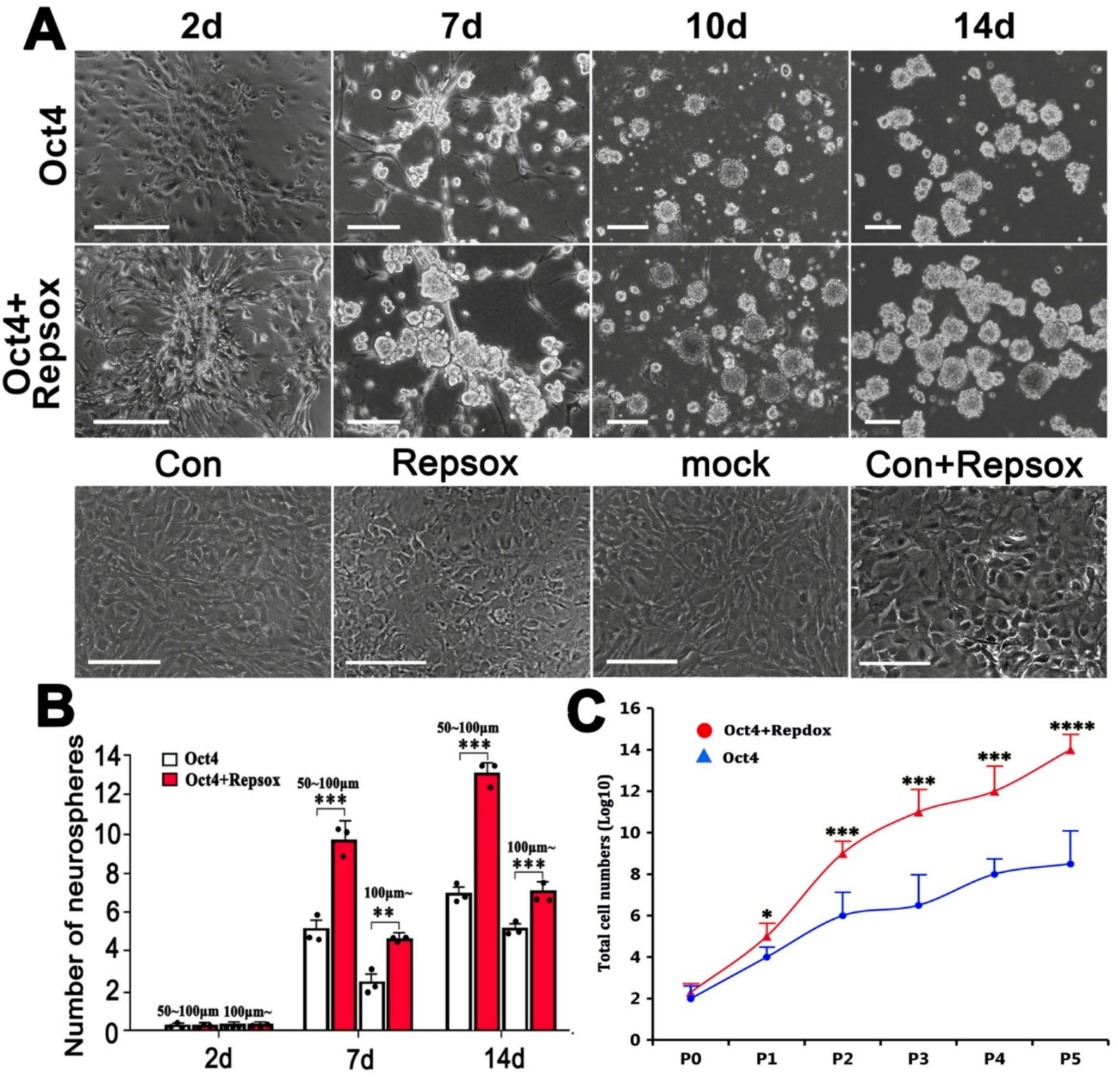
Generation of neurospheres from Oct4-transduced astrocytes with and without Repsox treatment. (**A**) Representative images of neurosphere formation in Oct4-transduced astrocyte cultures untreated (upper row of panels) or treated with Repsox (middle row of panels) at indicated time points. Scale bars: 100 μm. Notably, Con: Untransduced astrocytes cultured in NSC medium; Repsox: Untransduced astrocytes cultured in NSC medium supplemented with Repsox; mock: Empty vector-transduced astrocytes cultured in NSC medium; Repsox+mock: Empty vector-transduced astrocytes cultured in NSC medium supplemented with Repsox. (**B**) Quantitative analysis of neurospheres categorized by maximum diameter ranging from 50 μm to 100 μm and > 100 μm (*n* = 3 independent experiments). Note: Repsox treatment significantly increased neurosphere both the number and size distribution of neurospheres. (**C**) Growth curve of neurosphere cultures derived from Oct4-transduced astrocytes with or without Repsox treatment. Total cell numbers (Log10) are shown across 5 passages. Data are from 5 passages and represent means of 3 independent experiments. *p < 0.05 and ***p < 0.001 vs. their corresponding controls

To investigate the conversion of astrocytes into iNSCs, rat astrocytes were transduced with a lentiviral vector encoding Oct4, either alone or combined with Repsox. Morphological changes were subsequently monitored by phase-contrast microscopy, followed by quantitative assessment of sphere-forming capacity. As shown in Fig. 3A, Oct4 transduction alone triggered a pronounced morphological transformation: astrocytes progressively transitioned from a flat, confluent polygonal monolayer into clustered structure. Notably, within seven days post-transduction, cell clusters of various sizes progressively emerged and expanded, closely resembling neurospheres. By day 10, most aggregates had completely detached and formed free-floating neurospheres morphologically similar to those derived from primary NSCs (Fig. 3A-upper row of panel). Strikingly, the addition of Repsox to the Oct4-mediated astrocyte reprogramming system substantially enhanced its efficiency at parallel time points. Compared to those subjected to Oct4 transduction alone, the cells exhibited accelerated morphological transition kinetics, generated more neurospheres with significantly larger diameters, and showed enhanced expansion within spheres (Fig. 3A, middle row of panels). In contrast, untreated astrocytes, mock-transfected controls, and Repsox-only treated groups (both mock-transfected and untransfected) showed minimal morphological changes and negligible neurosphere formation (Fig. 3A, lower panels). These results indicate that Repsox significantly potentiates Oct4-mediated astrocyte reprogramming. To further examine the role of Repsox in the self-renewal capacity of Oct4-induced neurospheres, we cultured two distinct neurosphere types (derived from Oct4-transduced astrocytes) with or without Repsox for 14 days. Meanwhile, we systematically monitored neurosphere formation and growth, and quantified neurospheres across size categories (50–100 μm and > 100 μm) along with total cell counts from passages P0 to P5. Repsox+Oct4 treatment (50–100 μm: M = 0.19, SD = 0.038 for 2d, M = 9.31, SD = 0.87 for 7d, and M = 13.13, SD = 0.52 for 14d; >100 μm: M = 0.24, SD = 0.03 for 2d, M = 4.28, SD = 0.19 for 7d, and M = 7.13, SD = 0.21 for 14d) resulted in significantly higher numbers of neurospheres in both size categories compared to Oct4-transduced cultures (50–100 μm: M = 0.21, SD = 0.03 for 2d, M = 5.12, SD = 0.25 for 7d, and M = 6.75, SD = 0.18 for 14d; >100 μm: M = 0.214, SD = 0.03 for 2d, M = 2.27, SD = 0.23 for 7d, and M = 5.32, SD = 0.13 for 14d, *F*(1, 13) = 1.27, *p =* 0.62, *F*(1, 13) = 18.58, *p <* 0.001, *F*(1, 13) = 20.44, *p <* 0.001, *F*(1, 13) *=* 0.028, *p =* 0.87, *F*(1,13) = 11.80 *p <* 0.01, and *F*(1,13) = 17.12, *p* < 0.001, respectively) (Fig. 3B). Furthermore, although cell numbers increased progressively from P1 to P5 in all groups, Repsox-treated neurospheres (P0-P5: M = 1.98, SD = 0.47, M = 4.01, SD = 0.23, M = 6.03, SD = 1.09, M = 6.25, SD = 1.74, M = 7.92, SD = 0.84, M = 8.31, SD = 0.81) yielded significantly higher total cell counts relevant to untreated controls (P0-P5: M = 2.13, SD = 0.45, M = 4.85, SD = 0.75, M = 9.12, SD = 0.78, M = 11.03, SD = 1.08, M = 12.21, SD = 1.78, M = 13.85, SD = 1.82, *F*(1,3) = 0.021, *p* = 0.89, *F*(1,3) = 10.13, *p* = 0.049, *F*(1,3) = 78.50, *p <* 0.001, *F*(1,3) = 84, *p <* 0.001, *F*(1,3) = 87, *p <* 0.001, and *F*(1,3) = 76, *p <* 0.001, respectively) (Fig. 3C).

### Morphology and Cellular Characteristics of iNSCs

**Fig. 4:**
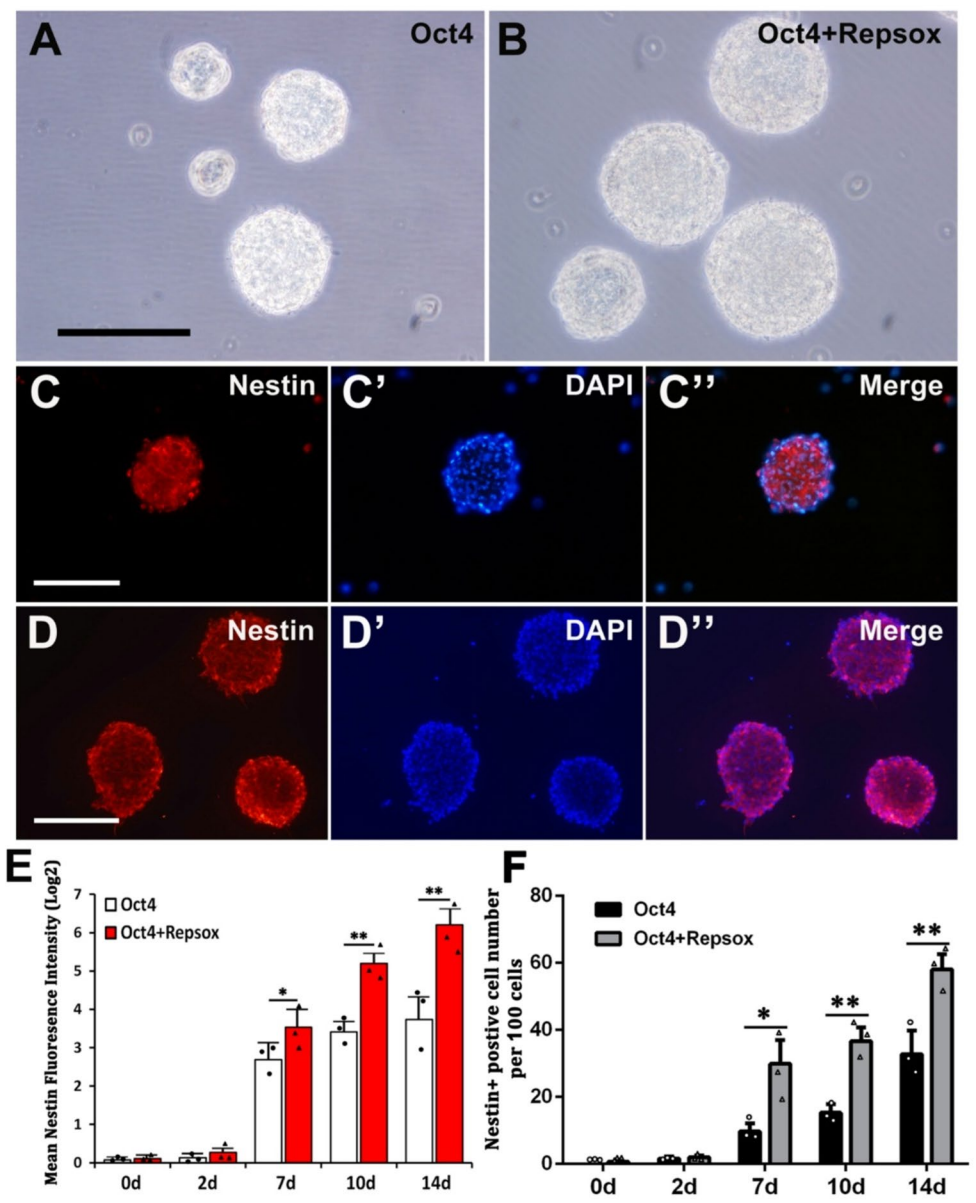
Characterization of induced neurospheres derived from rat astrocytes. The cells exhibited neurosphere morphological characteristics after transduction with Oct4 alone (**A**) or with Oct4 transduction with Repsox treatment (**B**). (**C**─**C’’**) and (**D**─**D’’**) Representative immunofluorescence images of neurospheres stained for Nestin (green) and counterstained with DAPI (blue) between days 10 and 14 following transduction with the Oct4, either without (**C**─**C’’**) or with (**D**─**D’’**) Repsox treatment. (**E**) Quantification of Nestin fluorescence intensity. Data represent mean ± SD; *n* = 3 independent experiments, *p < 0.05, **p < *0.01* and ***p < 0.001. (**F**) Quantitative analysis of cell numbers generated per 100 Nestin^+^ cells post-treatment with either Oct4 alone or Oct4/Repsox at the indicated time points. Note: Oct4/Repsox significantly enhanced proliferative capacity compared to Oct4 alone at all time points. Data represent mean ± SD, Data were obtained from three independent experiments (*n* = 3). For each experiment, 15 randomly selected fields were analyzed); *p < 0.05 and **p < *0.01.* Scale bars: 100 μm

To validate whether neurospheres derived from astrocytes acquired the characteristics of authentic NSCs, we systematically analyzed their morphology and biochemical phenotype, and Nestin expression dynamics at 14 days post-transduction, with or without Repsox. The results showed a progressive increase in Nestin expression in reprogrammed neurospheres following Oct4 transduction, irrespective of Repsox treatment. Notably, neurospheres co-stimulated with Repsox exhibited significantly greater number and larger sizes of Nestin-positive structures compared to those without Repsox (Fig. 4A-D). In line with the morphological and immunostaining data, quantitative analysis revealed that the addition of Repsox into Oct4 transduced cells resulted in a significant, time-dependent increase in both Nestin fluorescence intensity (M = 0.16, SD = 0.023, M = 0.31, SD = 0.028, M = 3.55, SD = 0.43, M = 5.25, SD = 0.24, M = 6.21, SD = 0.44) and the proportion of Nestin-positive cells (M = 0.02, SD = 0.001, M = 2.65, SD = 0.033, M = 31.21, SD = 9.15, M = 37.58, SD = 4.50, M = 59.70, SD = 4.43), compared to cells transduced with Oct4 alone (Nestin fluorescence intensity: M = 0.08, SD = 0.034, M = 0.163, SD = 0.029, M = 2.75, SD = 0.35, M = 3.32, SD = 0.030, M = 3.75, SD = 0.78, *F*(1,3) *=* 0.39, *p =* 0.85, *F*(1,3) *=* 0.23, *p =* 0.65, *F*(1,3) *=* 10.80, *p =* 0.035, *F*(1,3) *=* 26.50, *p <* 0.01, *F*(1,3) *=* 31.00, *p <* 0.01) and (the number of Nestin-positive cells: M = 0.018, SD = 0.001, M = 2.50, SD = 0.23, M = 9.25, SD = 1.85, M = 17.52, SD = 2.95, M = 32.25, SD = 8.71, *F*(1,3) *=* 0.10, *p =* 0.76, *F*(1,3) *=* 0.024, *p =* 0.88, *F*(1,3) *=* 12.50, *p =* 0.022, *F*(1,3) *=* 34, *p <* 0.01, *F*(1,3) *=* 31.00, *p <* 0.01) at all three time points examined (Fig. 4E and F).

### Biochemical and Phenotypic Traits of Oct4-Transduced Astrocytes with Repsox

**Fig. 5:**
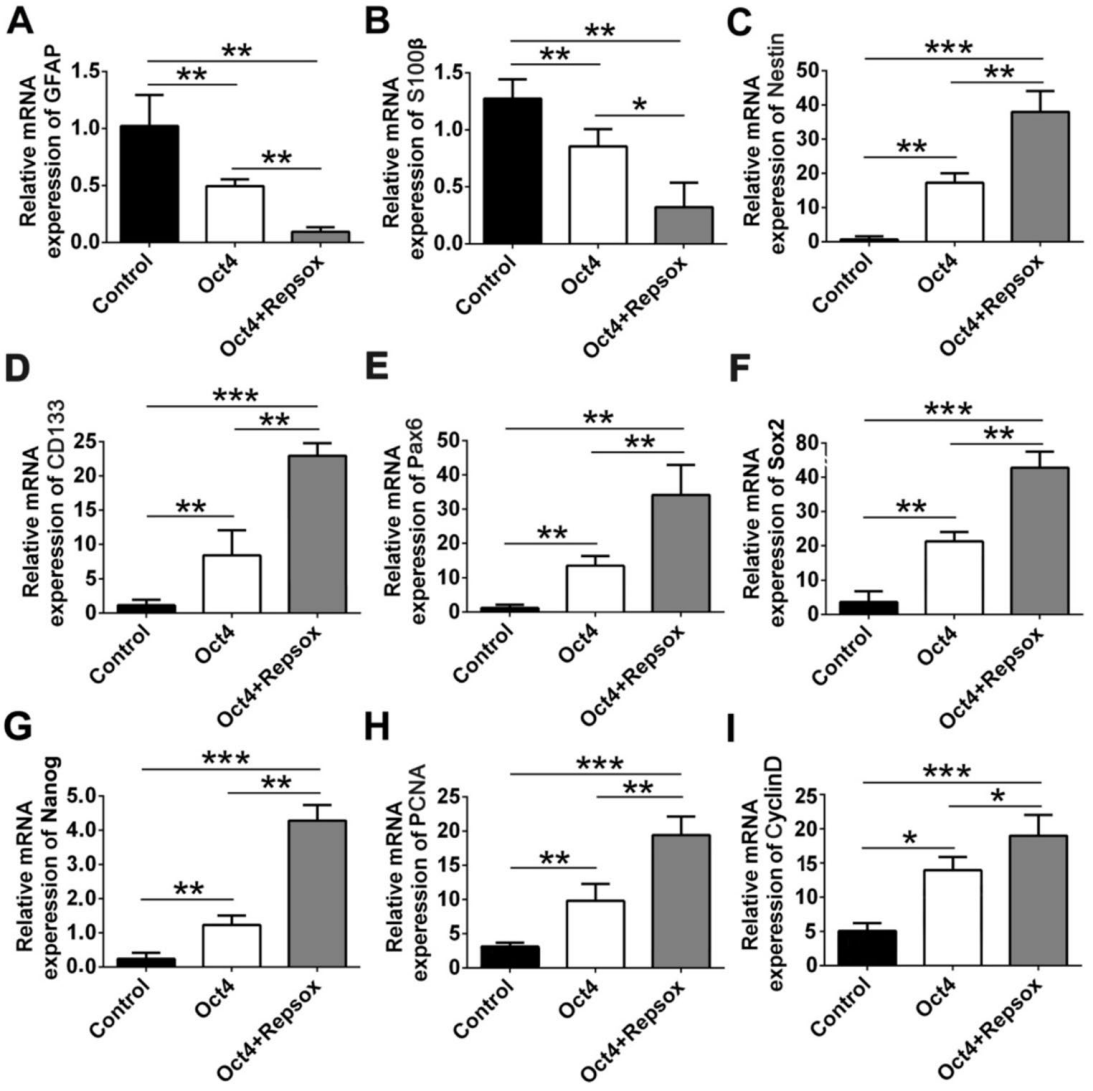
Gene expression profiles of astrocytes following Oct4 transduction with or without Repsox treatment. qRT-PCR analysis showed the transcript levels of astrocyte markers GFAP (**A**) and S100β (**B**); neural stem cell markers Nestin, Pax6, Sox2, CD133, and Nanog (**C**–**G**); and the cell proliferation markers PCNA and CyclinD1 (**H**,** I**) in Oct4-transduced astrocytes with or without Repsox for 10 days. GAPDH was used as the loading control for total RNA. Data represent mean ± SD from at least three biological replicates, five replicate wells were analyzed, and the resulting average value per experiment was used for statistical analysis; *p < 0.05, **p < 0.01 and ***p < 0.001 vs. the corresponding control, respectively. Notably, Con: Astrocytes in cultured NSC medium; Oct4: Oct4-transduced astrocytes cultured in NSC medium; Oct4/Repsox: Oct4-transduced astrocytes cultured in NSC medium supplemented with Repsox

To systematically evaluate the enhancing effect of Repsox’s on Oct4-mediated astrocyte reprogramming, we examined the mRNA levels of a subset of key characteristic markers for NSCs and astrocytes. As shown in Fig. 5, quantitative RT-PCR analysis within 10 days following Oct4 transduction showed that the expression of astrocyte-specific markers GFAP and S100β was progressively downregulated, both with and without Repsox (Fig. 5A and B). In contrast, mRNA levels of selected NSC markers Nestin, Pax6, CD133, Sox2, and Nanog, as well as the stemness associated markers PCNA and Cyclin D1 were progressively upregulated (Fig. 5C-I). Notably, the combination of Repsox treatment with Oct4 transduction produced a synergistic effect, resulting in more pronounced changes in the mRNA levels of all examined genes compared to Oct4-transduced alone.

### The Multipotency of iNSCs

**Fig. 6:**
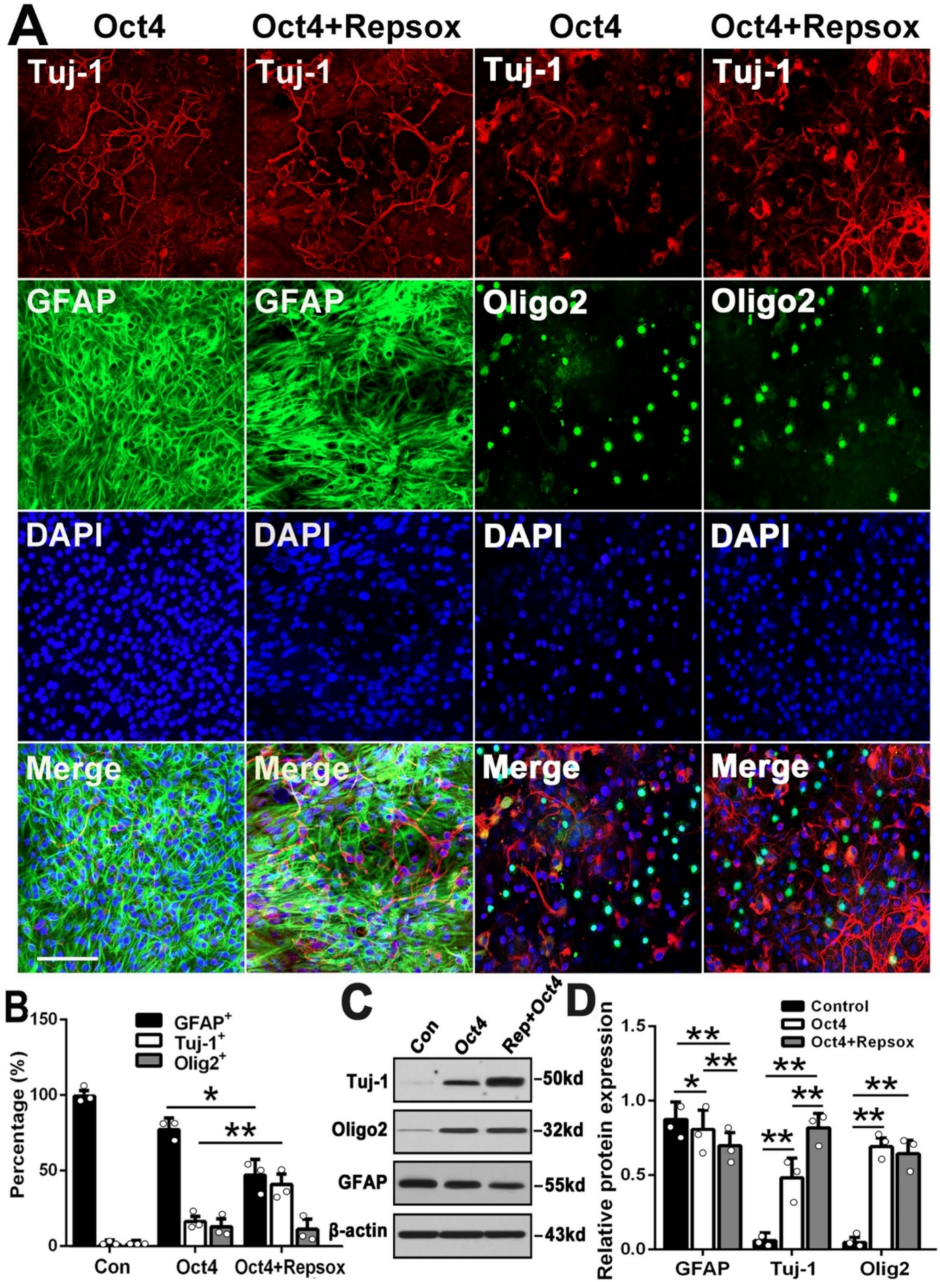
Tri-lineage differentiation potential of iNSCs reprogrammed from Oct4-transduced astrocytes with Repsox treatment. (**A**) Immunofluorescence of neurospheres following 10 days of differentiation into neurons, astrocytes, and oligodendrocytes. Tuj-1^+^ (red, arrows) neurons, GFAP^+^ (green) astrocytes, Oligo2^+^ (green) oligodendrocytes, and DAPI (blue) nuclei are shown. (**B**) Quantitative analysis of cell differentiation in neurospheres from the indicated groups. Data represent percentage of Tuj-1^+^, GFAP^+^, or Oligo2^+^ cells relative to total DAPI^+^ cells (mean ± SD). Notably 15 randomly selected fields were used for statistical analysis. (**C**) Western blot analysis of neural lineage markers in differentiated cells under the specified conditions. β-actin served as a loading control. (**D**) Densitometric quantification of neural lineage marker expression (Tuj-1, Oligo2, and GFAP) normalized to β-actin. Notably, Tuj-1 expression was significantly higher in iNSCs derived from Oct4/Repsox-treated astrocyte group than in those from Oct4-alone group. Notably, Con: astrocytes cultured in differentiation medium; Oct4: iNSCs derived from Oct4-transduced astrocytes in differentiation medium; Oct4/Repsox: iNSCs derived from Oct4-transduced astrocytes treated with Repsox in differentiation medium. Statistical significance: *p < 0.05, **p < 0.01 vs. Con group (one-way ANOVA with Tukey’s test). Data represent the means ± SD from at least three biological replicates

To evaluate the multipotency of astrocyte-derived iNSCs generated through Oct4 transduction with or without Repsox stimulation, we subjected them to spontaneous differentiation of iNSCs followed by immunofluorescence staining for three major neural lineages: neurons (Tuj-1), astrocytes (GFAP), and oligodendrocytes (Oligo2). Immunostaining demonstrated that iNSCs can successfully differentiated into Tuj1^+^ neurons, GFAP^+^ astrocytes, and Oligo2^+^ oligodendrocytes. Compared to Oct4 alone, Oct4 + Repsox-induced iNSCs yielded significantly more Tuj-1^+^ neurons fewer GFAP^+^ astrocytes, while Olig2^+^ oligodendrocytes were rarely observed in both groups, with no significant difference between the two groups (Fig. 6A). Quantitative analysis at day 9 post-differentiation revealed that iNSCs derived from Oct4/Repsox-treated astrocytes contained a higher percentage of Tuj-1^+^ cells (M = 38.35%, SD = 6.23) and a lower percentage of GFAP^+^ cells (M = 47.13%, SD = 8.61), compared to those derived from Oct4-transduced alone (M = 14.20%, SD = 2.16, *F*(1,3) *=* 38.5, *p <* 0.01; M = 14.20%, SD = 2.16, *F*(1,3) *=* 12.5, *p =* 0.024). Notably, the proportion of Oligo2^+^ cells was relatively higher in the Oct4-induced group (M = 11.70%, SD = 3.08) compared to Oct4/Repsox-induced groups (M = 9.40%, SD = 2.35). However, no statistical difference in the percentage of Oligo^+^ cells was observed (*F*(1,3) *=* 5.50, *p =* 0.084) (Fig. 6B). Western blot analysis supported these findings (Fig. 6C). Densitometric quantification revealed significantly higher protein level of the neuronal marker Tuj-1 in the Oct4/Repsox group (M = 0.76, SD = 0.68), concomitant with a marked reduction in the astrocytic marker GFAP (M = 0.68, SD = 0.077), compared to the Oct4-only group (M = 0.48, SD = 0.088, *F*(2,3) *=* 42, *p =* 0.002; M = 0.74, SD = 0.086, *F*(2,3) *=* 23, *p =* 0.008). Strikingly, Oligo2 protein expression levels were comparable between the two groups (Fig. 6D). Statistical analysis confirmed that Oct4/Repsox co-treatment significantly increased Tuj-1 and decreased GFAP expression (*p < 0.05, **p < 0.01; Fig. 6D). Collectively, these results indicate that combining Oct4 expression with Repsox treatment robustly enhances the reprogramming of astrocytes into functional, multipotent NSCs.

To further substantiate whether transplantation of NSCs derived from astrocytes can effectively differentiation into neurons, we performed immunofluorescence staining of spinal cord frozen Sections four weeks post-transplantation. The results demonstrated that in the transplantation of NSCs derived from Oct4/Repsox-treated astrocyte group, numerous Tuj-1/hoechest 33342 double-labeled cells were observed dispersing and migrating extensively away from the injection sites (Figure S1A) In contrast, in the group transplanted with NSCs derived from astrocytes treated with Oct4, the number of Tuj-1/hoechest 33342 double-labeled cells was relatively lower; however, the cell still exhibited a relatively broad migration pattern, with double-labeled cells detectable both within and beyond migration area is relatively extensive even though Tuj-1/hoechest 33342 double-labeled cells can also be seen in the transplantation region (Figure S1B). In the control transplantation group (astrocyte transplantation alone, no Tuj-1/hoechest 33342 double-labeled cells were detected either at the injection sites or in regions distant from them, a result consistent with that observed in the Repsox-treated astrocyte transplantation group (Figure S1C and S1D, respectively).

### Functional Neuronal Properties in Astrocyte-Derived Cells

**Fig. 7:**
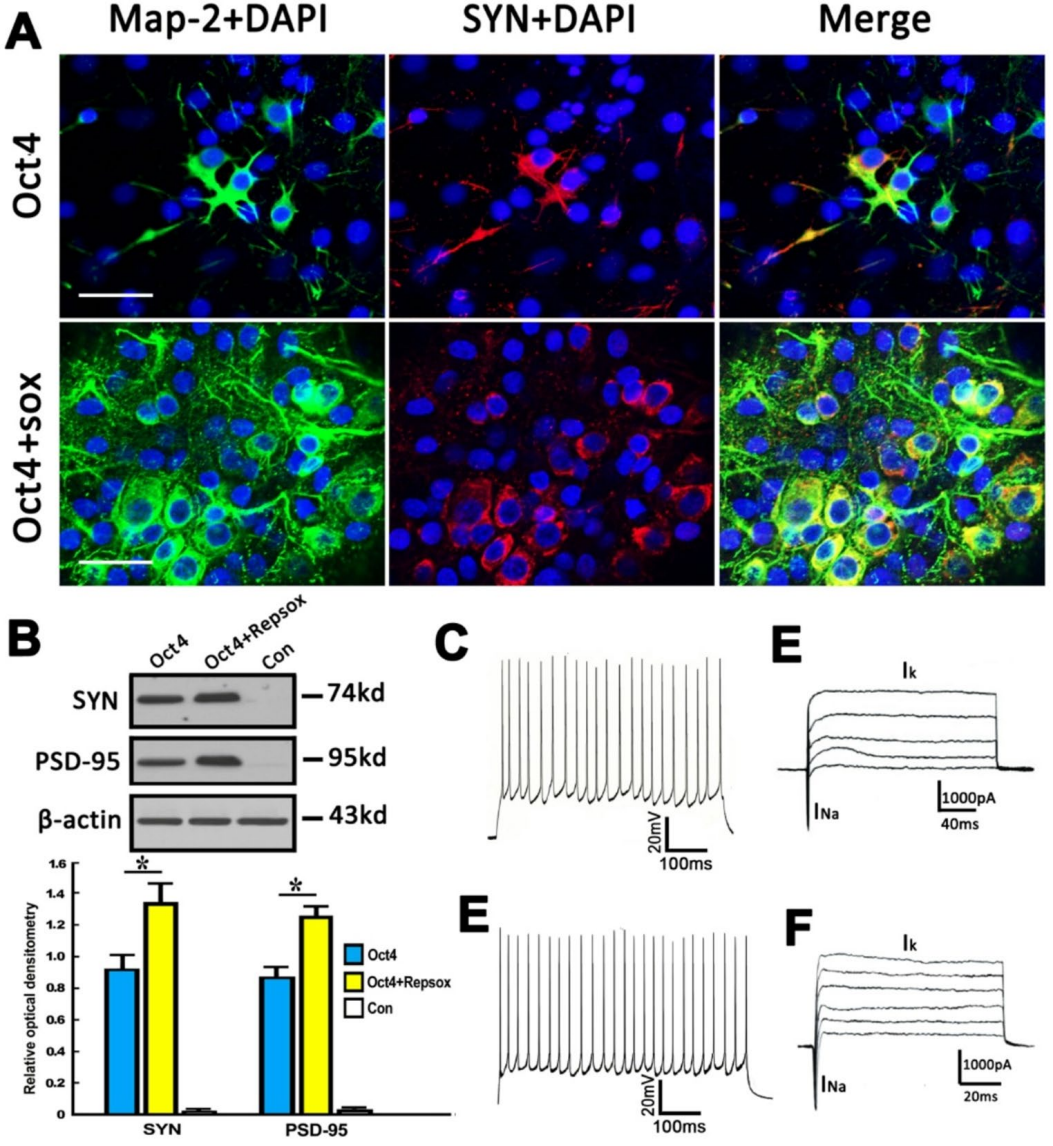
Functional characterization of neurons derived from astrocyte-reprogrammed iNSCs. (**A**) Representative immunofluorescence images of iNSC-derived neurons at three weeks, showing expression of synapsin (Red) and Map-2 (green). Scale bars: 50 μm. (**B**) Western blot analysis (upper panel) and densitometric quantification (lower panel) of synapsin and PSD-95 protein levels in iNSC-derived neurons. β-actin serves as a loading control for the total proteins. Data are representative of at least three biological replicates (*n* = 3). (**C**) Representative spontaneous action potentials recorded from Oct4-induced neurons, displaying irregular firing patterns with variable amplitudes. (**D**) Representative whole-cell patch clamp recordings of voltage-gated Na⁺ and K⁺ currents in Oct4-induced neurons. (**E**) Neurons derived from Oct4/Repsox-induced NSCs exhibited mature electrophysiological characteristics, including regular spontaneous action potentials with consistent amplitude, resembling native neuronal firing patterns. (**F**) Representative whole-cell recordings showing robust voltage-dependent Na⁺/K⁺ currents in differentiated neurons derived from Oct4/Repsox-induced NSCs

To assess the functional maturity of astrocyte-derived neuron-like cells, we first conducted double immunostaining for MAP-2 and synapsin (SYN, a specific marker for functional neurons) together with western blot analysis of SYN and PSD-95 expression. As shown in Fig. 7A, MAP2^+^ cells exhibited pronounced synapsin immunoreactivity with a characteristic punctate pattern staining in both soma and neurites, which was undetectable in control samples (data not shown). The synapsin signal intensity was remarkedly stronger in neurons derived from the Oct4/Repsox group than that in the Oct4-only group. Consistently, western blot analysis revealed that the protein levels of both synapsin (M = 1.360, SD = 0.109) and PSD-95 (M = 1.310, SD = 0.035) were significantly elevated in reprogrammed cells relative to Oct4-only groups (M = 0.915, SD = 0.065, *F*(2,3) *=* 8.50, *p =* 0.038; M = 0.872, SD = 0.045, *F*(2,3) *=* 10.50, *p =* 0.026), with the highest expression observed in the Oct4/Repsox group (Fig. 7B).

Next, we examined the electrophysiological properties of the converted neurons following 3 weeks of differentiation. As shown in Fig. 7C and E, under current-clamp conditions, a subset of the reprogrammed neurons (5 out of 8 recorded) fired repetitive action potentials. Notably, neurons converted by Oct4 alone exhibited irregular spontaneous spikes (Fig. 7C), indicative of neuronal functionality. In contrast, those neurons derived from the Oct4/Repsox group displayed regular spiking patterns with increased action potential amplitude (Fig. 7E). Voltage-clamp recordings further confirmed the presence of voltage-gated Na^+^ and K^+^ currents in both groups, indicative of neuronal functionality. Importantly, current amplitudes were significantly larger in Oct4/Repsox-converted neurons (Fig. 7D) than in Oct4-only counterparts (Fig. 7F), indicating enhanced functional maturity.

### The Notch1/Hes1/Smurf2 Pathways Orchestrates Repsox’s Enhancement of Oct4-Driven Reprogramming of Astrocytes into NSCs

**Fig. 8:**
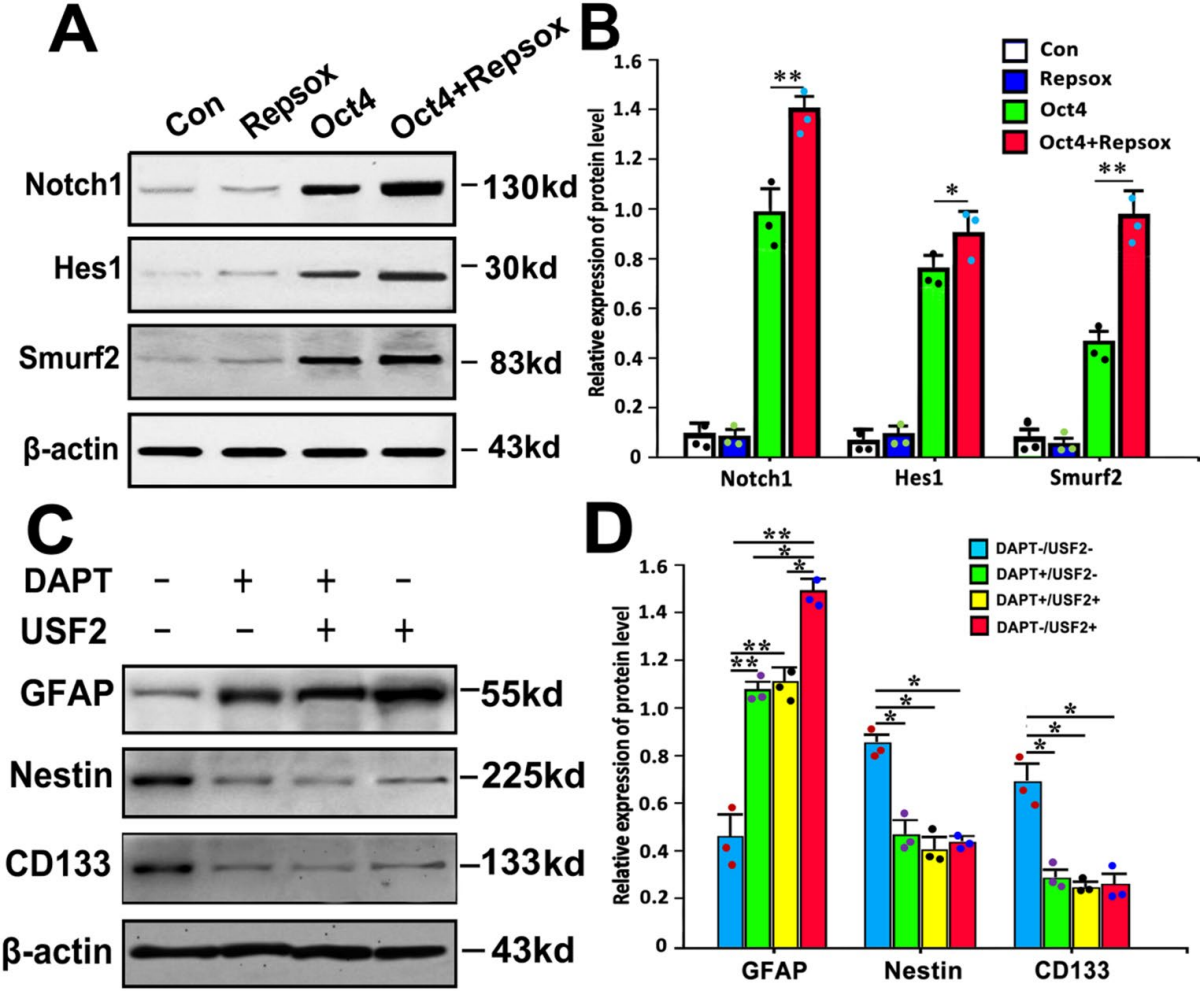
Repsox enhances Oct4-driven astrocyte reprogramming via Notch1/Hes1/Smurf2 axis. (**A**) Representative western blots showing protein levels of total Notch1, Hes1, and Smurf2 in astrocytes after 10 day treatments. β-actin served as a loading control. (**B**) Quantitative analysis of the relative protein levels of Notch1, Hes1, and Smurf2 normalized to β-actin. (*n* = 3 per group). Data are presented as means ± SD from at least three biological replicates (*n* = 3). *p < 0.05, **p < 0.01, and ***p < 0.001. (**C**) Western blot analysis of NSC marker expression in reprogrammed astrocytes treated with pathway inhibitors: DAPT (a Notch1 antagonists) or USF2 (a Smurf inhibitor). (**D**) Quantification of GFAP, Nestin and CD133 expression normalized to β-actin. Data are presented as means ± SD from at least three biological replicates (*n* = 3). Note that both selective inhibitors significantly suppressed Oct4/Repsox-mediated astrocyte-to-NSC reprogramming. *p < 0.05 and **p < 0.01. Notably, Con, astrocytes cultured in NSC medium; Oct4, Oct4-transduced astrocytes cultured in NSC medium; Oct4/Repsox, Oct4-transduced astrocytes cultured in NSC medium supplemented with Repsox

Although our data demonstrate that Repsox effectively enhances Oct4-driven astrocytes-to-NSC reprogramming, the underlying molecular mechanisms remain largely unknown. Given the key roles of Notch1/Hes1 signaling in stemness maintenance and of Smurf2 in cell cycle and epigenetic regulation, we postulated their potential involvement. Western blot analysis revealed that Oct4 transduction alone significantly increased protein levels of Notch1, Hes1, and Smurf2 compared to controls. Notably, the addition of Repsox further significantly potentiated the expression of all three molecules (Fig. 8A). Quantitative densitometry of the immunoblots showed that the combination of Oct4 and Repsox (Notch1: M = 1.409, SD = 0.05, Hes1: M = 0.901, SD = 0.11, and Smurf2: M = 0.965, SD = 0.049) significantly increased protein expression compared to Oct4 transduction alone (Notch1: M = 0.982, SD = 0.12, *F*(3,3) *=* 23.50, *p =* 0.006; Hes1: M = 0.770, SD = 0.087, *F*(2,3) *=* 9.30, *p =* 0.045, and Smurf2 M = 0.425, SD = 0.018 *F*(3,3) *=* 24.50, *p =* 0.0057) (Fig. 8B), suggesting that the activation of the Notch1/Hes1/Smurf2 signaling pathways may contribute to Repsox-enhanced Oct4-driven astrocyte reprogramming.

To functionally substantiate the role of this pathway in Repsox-mediated enhancement of Oct4-driven astrocyte reprogramming, astrocytes were pretreated with several selective inhibitors targeting its key components, DAPT (a γ-secretase inhibitor that blocks Notch1 signaling) and USF2 (a Smurf2 inhibitor) prior to Oct4 transduction and Repsox stimulation. Western blot analysis showed that combined or individual inhibition of Notch1 and Smurf2 significantly reduced the expression of NSC markers Nestin (M = 0.458, SD = 0.038, *F*(3,3) *=* 11.50, *p =* 0.028, M = 0.401, SD = 0.038, *F*(3,3) *=* 10.80, *p =* 0.033, M = 0.425, SD = 0.019, *F*(3,3) *=* 12.50, *p =* 0.023) and CD133 (M = 0.287, SD = 0.023, *F*(3,3) *=* 10.50, *p =* 0.034, M = 0.243, SD = 0.018, *F*(3,3) *=* 11.20, *p =* 0.029, M = 0.252, SD = 0.022, *F*(3,3) *=* 10.00, *p =* 0.037), compared to untreatment groups (M = 0.845, SD = 0.022, M = 0.685, SD = 0.065,) (Fig. 8C and D). Conversely, a marked upregulation of the astrocyte marker GFAP (M = 1.075, SD = 0.023, *F*(3,3) *=* 24.70, *p =* 0.0056, M = 1.128, SD = 0.022, *F*(3,3) *=* 21.80, *p =* 0.0077, M = 1.490, SD = 0.031, *F*(3,3) *=* 20.50, *p =* 0.0085) was observed after 2 days of inhibitor treatment compared to untreatment group (M = 0.467, SD = 0.089) (Fig. 8D). Collectively, these findings indicate that intact Notch1/Hes1/Smurf2 signaling is essential for Repsox to effectively enhance Oct4-mediated astrocyte-to-NSC conversion.

## Discussion

Neural stem cells (NSCs), characterized by their self-renewal capabilities and multipotent differentiation potential, have emerged as promising candidates for treating nerve injuries and neurodegenerative disorders by replacing degenerative or necrotic neurons (Taufer et al. 2020; Lin et al. 2021; Guo et al. 2021a, b, c; Mcintyre et al. 2021; De Gioia et al. 2020). Nevertheless, the clinical translation of NSC-based therapies faces a significant challenge due to their limited availability from conventional sources. Therefore, it is imperative to develop alternative strategies for generating desired cell types required for neural regeneration. Growing evidence indicates that cell reprogramming technology could provide a promising alternative strategy for producing desired cell types to circumvent the existing limitations of traditional cell sources (Guan et al. 2022; Zhao et al. 2020; Romanazzo et al. 2020; Takahashi et al. 2006).

In this study, we provide in vitro evidence that Repsox effectively enhances Oct4-transduced reprogramming of astrocytes into iNSCs. The iNSCs derived from astrocytes acquired key characteristics resembling those of authentic NSCs. First, they exhibit similar morphological features, rapidly forming free-floating neurospheres with robust self-renewal capacity. Secondly, the iNSCs share characteristic molecular phenotype, including the expression of authentic NSC markers. Thirdly, each passaged neurospheres maintained free-floating growth and expanded progressively in suspension culture over multiple passages. Lastly, similar to authentic NSCs, the iNSCs possessed the ability of multipotency to readily differentiate into neurons, astrocytes, and oligodendrocytes. Notably, the neurons derived from the reprogrammed astrocytes displayed functional neuronal characteristics, including synapse formation and electrophysiological activity. These findings indicate that the forced Oct4 expression combined with Repsox stimulation effectively converts astrocytes into iNSCs closely resembling native counterparts. Importantly, Repsox functions as a highly effective enhancer of Oct4-driven astrocyte-to-iNSC reprogramming, demonstrating high efficacy in the direct conversion of mature astrocytes into NSCs. Furthermore, astrocyte-derived iNSCs maintained neural lineage commitment without undergoing cell lineage switching, thus validating their neural functional specificity. More importantly, our data reveal that Repsox substantially augments Oct4-mediated astrocyte reprogramming by activating the Notch1/Hes1/Smurf2 signaling cascade, which orchestrates critical reprogramming events, including silence of astrocyte-specific genes, specification of NSC identity, promotion of self-renewal, and the maintenance of stemness.

Previous compelling studies have highlighted that TF-mediated cell reprogramming is one of the most predominantly favored and widely used strategies due to its relatively straightforward procedure and high conversion efficiency (Ichida et al. 2009; Yang et al. 2019; Takahashi et al. 2007; Okahara-Narita et al. 2012). Although various somatic and glial cells have been successfully reprogrammed into target lineages by enforcing specific TF expression (Kempf et al. 2021; Aravantinou-Fatorou et al. 2020; Heinrich et al. 2011), TF-based reprogramming is often plagued by inherent limitations, including cellular instability, low efficiency, and the generation of intermediate progenitor populations, which are largely attributable to technical complexity and suboptimal yields. A particularly critical challenge is the requirement to deliver multiple TFs simultaneously into the host cell genome. Consequently, the development of optimized strategies that enhance reprogramming efficacy with a minimal set of inductors is paramount importance. Accumulating evidence highlights Oct4 as a master regulator of cell-fate specification and pluripotency maintenance (Le Bin et al. 2014; Wang et al. 2014). Its upregulation typically initiates the activation of pluripotency-associated gene networks, sustains stem cell properties, and potentiates self-renewal capacity (Chambers et al. 2004). These studies highlight Oct4 unique molecular features indispensable for initiating cell fate transitions during reprogramming. This process frequently involves Sox2, another essential TF that is upregulated and activated in somatic cell reprogramming (Okahara-Narita et al. 2012; Kempf et al. 2021; Aravantinou-Fatorou et al. 2020). Notably, Sox2 selectively interacts with Oct4 at canonical binding sites to form a heterodimeric Oct4/Sox2 complex. This partnership orchestrates the expression of pluripotency and self-renewal genes, thereby promoting cell-cycle re-entry and lineage acquisition (Mansour et al. 2013; Aksoy et al. 2013), indicating that Sox2-mediated signaling directly contributes to the transcriptional activation of NSC intrinsic genes. Repsox, a small-molecule inhibitor of TGF-β signaling, has been shown to functionally substitute for Sox2 by promoting Nanog expression during reprogramming (Ichida et al. 2009; Woltjen et al. 2009; Gancheva et al. 2024). This substitution potentially circumvents the technical challenges associated with exogenous gene integration, and mitigates risks of viral insertional mutagenesis. Moreover, Repsox enhances induced pluripotent stem cell (iPSC) generation by inhibiting TGF-β signaling pathway (Deng et al. 2015; Zhou et al. 2018; Qin et al. 2019). Additionally, Repsox has been shown to improve the developmental potential of interspecies somatic cell nuclear transfer embryos through the modulation of pluripotency-associated gene expression (Zhu et al. 2017). Collectively, these findings position Repsox as a substantial potential in regulating core genetic programs governing cell fate.

In comparison to conventional virus-mediated techniques, small-molecule-based approaches provide a more convenient, efficient, rapid, and safer strategy for triggering cellular reprogramming, which may facilitate future clinical translation. Although certain somatic cells, such as fibroblasts and endothelial cells, are relatively accessible and offer considerable advantages like higher yields and greater proliferative capacity during reprogramming, they are not of neural origin. Consequently, lineage switching is inevitable during reprogramming, which potentially compromises their ability to form functional synapses and integrate neuronal circuits (Zhu et al. 2015; Yang et al. 2011). By contrast, astrocytes emerge as a highly promising candidate for reprogramming because they can circumvent lineage commitment switching, thereby improving the efficacy of cell-based therapies. Previous studies have shown that TF-mediated reprogramming typically relies upon multiple TFs and is hampered by technical complexity, time-consuming, tumorigenic risk, and epigenetic instability (Rivetti et al. 2017; Yang et al. 2019). To overcome these limitations, we developed a novel approach for generating NSCs from mature astrocytes utilizing only a single neural-progenitor-inducing factor, Oct4, in combination with Repsox. By minimizing the number of exogenous IFs, this strategy substantially reduces the risk of viral insertional mutagenesis and tumorigenesis. As a result, it offers a relatively safe and potentially innovative cell-based therapeutic avenue for CNS injuries.

Our previous work demonstrated that mature astrocytes can be directly reprogrammed into iNSCs using single transcription factor Oct4 (Yang et al. 2019). However, the efficiency of Oct4-mediated reprogramming remains suboptimal compared to approaches employing multiple factors. In this study, we reveal that Repsox significantly enhances the efficiency of Oct4-driven astrocyte-to-NSC conversion. This enhancement was evidenced by accelerated morphological changes, increased proliferation, and a shorter time to acquire NSC properties. These findings were further substantiated by expression of NSC biochemical phenotypic markers (e.g., Nestin, PAX6, CD133, Sox2, and Nanog) and stemness-related hallmarks (PCNA and cyclin D1) at the higher levels, together with a concomitant reduction of astrocyte markers (GFAP and S100β), compared to Oct4 only-mediated reprogramming. This effect may be attributed to the unique properties of Repsox to stimulate NSC-specific gene expression while repressing differentiation signals, thereby facilitating the transition to an undifferentiated state. Alternatively, Repsox could be indispensable for modulating Oct4 enhancers that govern multipotency and self-renewal genes during astrocyte reprogramming. Consistent with prior reports in fibroblast-to-iPSC reprogramming, Repsox may specifically substitute for Sox2 by inhibiting TGF-β signaling and activating endogenous pluripotency-associated genes during the reprogramming of fibroblasts (Ichida et al. 2009; Woltjen et al. 2009; Mu et al. 2015; Huangfu et al. 2008). Besides, Sox2 itself functions as a critical regulator of self-renewal and pluripotency in various stem cell types (Mu et al. 2015; Han et al. 2013; Huangfu et al.^ 2008^). Based on the reports, we speculated that Repsox similarly functions to replace Sox2 during astrocyte-to-NSC reprogramming. In addition to Sox2, Nanog plays a key role in maintaining embryonic stem cells in an undifferentiated state (Chambers et al. 2003) and has been demonstrated to augment reprogramming efficiency (Suzuki et al. 2006; Xu et al. 2008). In our experiments, Nanog expression was significantly higher in Repsox-treated group than with Oct4 alone. This suggests that the upregulated Nanog may compensate for the absence of Sox2 during Oct4-driven astrocyte reprogramming, thereby amplifying pluripotency-associated transcriptal program.

**Fig. 9:**
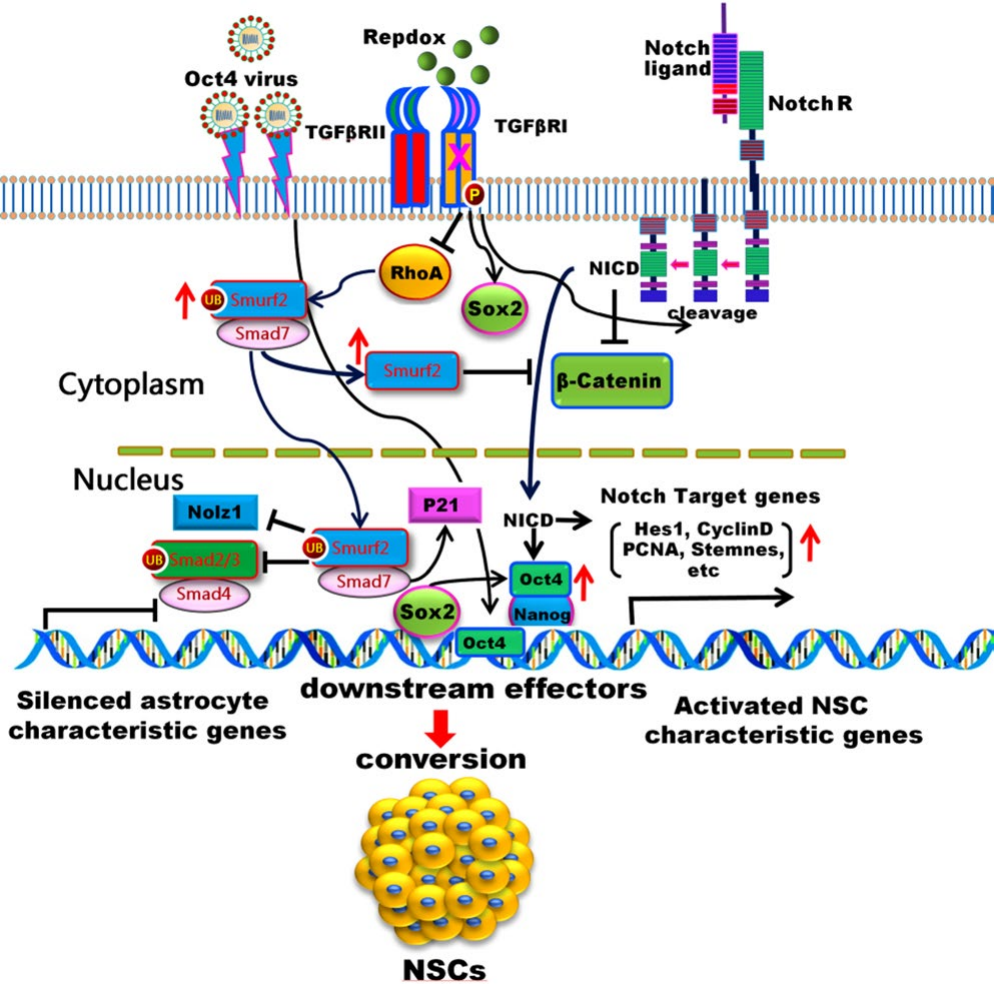
A schematic diagram illustrating the underlying molecular mechanism through which Repsox enhances Oct4-induced reprogramming of astrocytes into NSCs, involving the coordinated activation of the Notch1/Hes1/Smurf2 signaling axis. UP: Ubiquitination; P: phosphorylation

In this study, we demonstrate that Repsox effectively circumvented the requirement for transgenic expression of Sox2 or Nanog. Notably, molecular characterization of iNSC pluripotency, particularly their differentiation potential into neuronal and glial lineages in vivo and in vitro, revealed that iNSCs generated by Oct4 combined with Repsox exhibit a greater propensity for neuronal differentiation and a comparatively lower tendency for glial differentiation than those induced by Oct4 alone. This indicates that Oct4/Repsox-induced NSCs acquire several characteristics more closely resembling authentic NSCs, displaying a differentiation bias toward neuronal rather than glial progenitor fate. Furthermore, elucidating the molecular mechanism underlying astrocyte reprogramming remains a considerable interest. Our data demonstrate substantial upregulation of Notch1, its downstream effector Hes1, and Smurf2 in Oct4-transduced astrocytes treated with Repsox. This suggests that the Notch1/Hes1/Smurf2 signaling cascade plays a functional role in reprogramming, as the conversion of astrocytes into iNSCs was remarkably suppressed by co-treatment of the specific inhibitors DAPT and USF2. Given crucial roles of Notch1 and Smurf2 signaling in cell cycle re-entry, epigenetic modulation, cell proliferation, and cell fate commitment (Uribe-Etxebarria et al. 2017; Aguirre et al. 2010; Brandenberger et al. 2004; Lin et al. 2000), our findings strongly support that Repsox cooperates with Oct4 to reprogram astrocytes into NSCs primarily through activation of the Notch1/Hes1/Smurf2 signaling pathway (Fig. 9). Nevertheless, whether similar efficiency can be achieved in human cells remains unknown, highlighting the need for validation in human cell models.

**Table 1:** Primer genes used for RT-PCR

Genes	Forward primer	Reverse primer
*GFAP*	5’-GAGGGACAACTTTGCACAGG-3’	5’-GTCTATACGCAGCCAGGTTG-3’
*Olig2*	5’-GGCGGTGGCTTCAAGTCATC-3’	5’-TAGTTTCGCGCCAGCAGCAG-3’
*S100β*	5’- CTTGCTCAGCCTGCTTTCTT-3’	5’- GCCTTCTCCAGCTCAGA**CAT**-3’
*nestin*	5’-TGCAGCCACTGAGGTATCTG-3’	5’-CAGTTCCCACTCCTGTGGTT-3’
*CD133*	5’-CTTGGCATCGCGTTTGG-3’	5’-GAGCCCGCAAGTCTCTGTAATT-3’
*Sox2*	5’-GCAGTACAACTCCATGAC-3’	5’-GCGAGTAGGACATGCTGTAGGTG-3’
*Pax6*	5’-GGCAACCTACGCAAGATGGC-3’	5’-TGAGGGCTGTGTCTGTTCGG-3’
*Nanog*	5’-TCTCCTCCGCCTTCCTCT-3**’**	5’-TTGCCTCTGAAACCTATCCTTG-3’
*PCNA*	5’-TTGTCAGCAAGACCTCGCTC-3′	5′-CTGGGATTCCAAGTTGCTCA-3
*CyclinD1*	5’-CTCCCCACGATTTCATCGAA-3’	5’-GTGCATGTTTGCGGATGATC-3’
*GAPDH*	5’-GGTTGTCTCCTGCGACTTCA-3’	5’-TGGTCCAGGGTTTCTTACTCC-3’

## Conclusions

This study demonstrates that the transcription factor Oct4, in conjunction with small-molecule compound Repsox, efficiently reprogram rat astrocytes into iNSCs, albeit with some heterogeneity in their neural differentiation outcomes. This approach offers significant advantages over conventional multi-factor reprogramming strategies in terms of operational convenience, efficiency, and safety, while potentially circumventing risks associated with viral vector integration. Notably, the Oct4/Repsox combination achieves superior reprogramming outcomes, characterized by higher efficiency, greater expandability, and enhanced neurosphere-forming capacity. Furthermore, Oct4/Repsox-induced iNSCs displayed a stronger differentiation propensity toward neuronal lineages and reduced glial commitment compared to those induced by Oct4 alone. Although the precise molecular mechanism underlying Repsox/Oct4-meidated astrocyte reprogramming still needs to be further determined, our data strongly suggest that the activation of the Notch1/Hes1/Smurf2 signal axis plays a key role in this process, likely facilitating epigenetic silencing of GFAP gene and promoting maintenance of stem-cell properties. Despite these significant advances, several limitations should be acknowledged: (1) viral vector delivery of Oct4 still poses potential genotoxic risks; (2) long-term safety and functional integration of these iNSCs require validation in vivo; and (3) current findings from rodent models may not fully translate to human astrocyte reprogramming. Notwithstanding these limitations, this strategy demonstrates high efficiency and stability, providing a promising avenue for further exploration of NSC generation and enhancing the potential of autologous cell-based therapies for the treatment of CNS injuries and neurodegenerative disorders. Based on the present findings, future efforts should prioritize validating reprogramming in human cell models, adopting non-integrating delivery methods, improving homogeneous differentiation, and assessing therapeutic efficacy in relevant disease models.

## Supplementary Information

Below is the link to the electronic supplementary material.

Supplementary Material 1. Fig. 1 Neuronal differentiation of iNSCs after transplantation into the rat spinal cord. Immunofluorescence staining Tuj-1 illustrates the in vivo neuronal differentiations of transplanted NSCs derived from Oct4/Repsox-treated astrocytes (A1-2), Oct4-transduced astrocytes (B1-2), Repsox-treated astrocytes (C1-2), and normal astrocytes (D1-2). Notably, cells double-labeled positive for Tuj-1 and hoechst 33,342 correspond to transplanted NSCs that have differentiated neurons (arrows). Only Tuj-1 single-positive signals indicate endogenous neurons of the spinal cord (arrowheads), whereas Hoechest 33,342 labeling alone identifies transplanted exogenous cells, including non-neuronal cell types (asterisks).

## Data Availability

Not applicable.
