# Supplementary figures and images for: The Small Chemical Compound Repsox Potentiates Oct4-Driven Astrocyte-to-Neural Stem Cell Reprogramming via Notch1/Hes1/Smurf2 Pathway
Source: Cell Mol Neurobiol. 2026 Apr 24;46:99. doi: 10.1007/s10571-026-01731-9 (PMC13243170; doi:10.1007/s10571-026-01731-9)

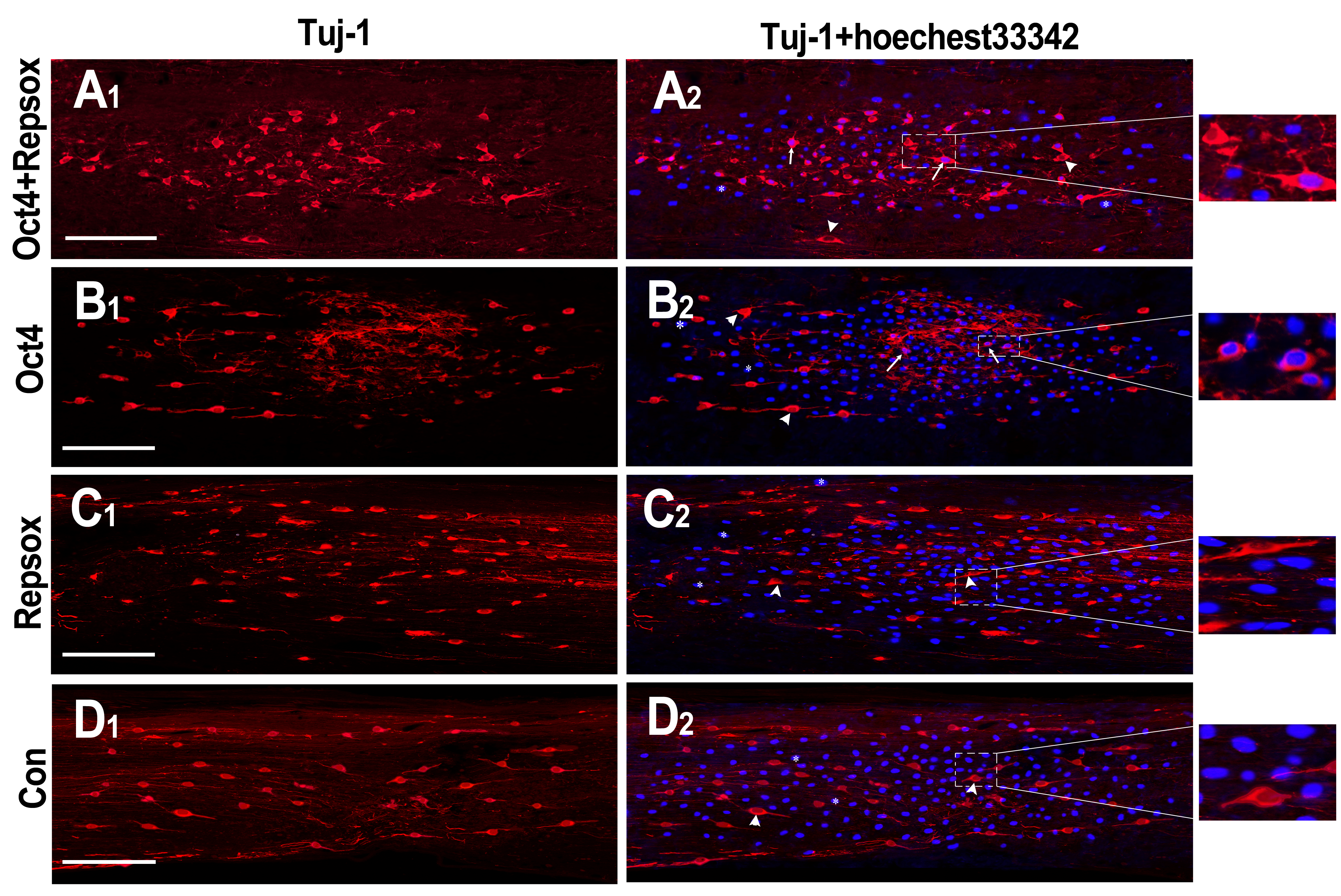

Supplement: Supplementary file 1 — Supplementary Material 1. Fig. 1 Neuronal differentiation of iNSCs after transplantation into the rat spinal cord. Immunofluorescence staining Tuj-1 illustrates the in vivo neuronal differentiations of transplanted NSCs derived from Oct4/Repsox-treated astrocytes (A1-2), Oct4-transduced astrocytes (B1-2), Repsox-treated astrocytes (C1-2), and normal astrocytes (D1-2). Notably, cells double-labeled positive for Tuj-1 and hoechst 33,342 correspond to transplanted NSCs that have differentiated neurons (arrows). Only Tuj-1 single-positive signals indicate endogenous neurons of the spinal cord (arrowheads), whereas Hoechest 33,342 labeling alone identifies transplanted exogenous cells, including non-neuronal cell types (asterisks). [file 10571_2026_1731_MOESM1_ESM.tif]
